# Biologicals and bone loss

**DOI:** 10.1186/ar3722

**Published:** 2012-03-08

**Authors:** Willem F Lems

**Affiliations:** 1 VU University Medical Center, Amsterdam, The Netherlands

## 

Inflammatory joint diseases like rheumatoid arthritis (RA), as well as other rheumatic conditions such as ankylosing spondylitis and systemic lupus erythematosus, comprise a heterogeneous group of joint disorders that are all associated with extra-articular side effects, including bone involvement. Disease activity, immobility and treatment with glucocorticoids are the main factors that increase the risk of osteoporotic fractures, on top of the background fracture risk based on, amongst others, age, body mass index and gender. It is thought that the pathogenesis of both peri-articular and generalized osteoporosis and local bone erosions share common pathways. This hypothesis has been strengthened by the discovery that osteoclasts, stimulated mostly by the receptor activator of nuclear factor kappa B ligand (RANKL) pathway, play a central role in all of these processes.

Generalized bone loss has been documented in cross-sectional studies: a 2-fold increase in osteoporosis, defined as a T-score <-2.5 in females and Z-score <-1 was found in 394 postmenopausal RA-patients and 192 male RA-patients [1]. Before the introduction of biologicals, a high bone loss was also observed in a longitudinal study in early RA: -2.4% at the spine and -4.3% at the trochanter [2]. Against that background, it is relevant that we investigated whether treatment with anti-TNF-α prevents loss of bone mineral density at the spine and hip (generalized) and in the hands (local) in patients with rheumatoid arthritis (RA) and during anti-TNF treatment [3]. 102 patients with active RA, who were treated with infliximab during one year, were included into this open cohort study. The BMD of the spine and hip was unchanged during treatment with infliximab, whereas BMD of the hand decreased significantly by 0.8% (p < 0.001). The BMD of the hip in patients with an EULAR good response showed a favorable change compared with patients not achieving such a response. This is a proof that the usually occurring generalized bone loss in patients with RA can be arrested by the use of aggressive antirheumatic drugs, such as anti-TNF.

Next to BMD changes upon anti-TNF, we investigated the changes in bone markers, to elucidate the underlying mechanism of the favourable effect of anti-TNF. Bone formation was measured by osteocalcin (OC) and bone resorption was determined by b-isomerized carboxy terminal telopeptide of type 1 collagen (b-CTx); osteoclast regulating proteins including the soluble receptor activator of Nfκb (s-RANKL) and osteoprotegerin (OPG) were determined in serum using an ELISA from Immun-diagnostik. Serum β-CTx and RANKL were both significantly decreased compared to baseline at all time points. The decrease in β-CTx was associated to the decrease in DAS-28 and CRP during the 0 to 14 weeks interval. No changes were observed in serum osteocalcin and OPG. These data on bone mineral density emphasizes that the arrested bone loss at the spine and hips can be described to a large extent to a decrease in disease activity.

Later on, it was also shown that bone loss could be arrested in RA-patient treated with adalimumab [4], and the favourable effects of anti-TNF on bone markers were also observed during treatment with rituximab [5].
